# Overtreatment of COPD with Inhaled Corticosteroids - Implications for Safety and Costs: Cross-Sectional Observational Study 

**DOI:** 10.1371/journal.pone.0075221

**Published:** 2013-10-23

**Authors:** Patrick White, Hannah Thornton, Hilary Pinnock, Sofia Georgopoulou, Helen P. Booth

**Affiliations:** 1 Department of Primary Care and Public Health Sciences, King’s College London, King’s Health Partners, London, United Kingdom; 2 Allergy and Respiratory Research Group, Centre for Population Health Sciences, University of Edinburgh, Edinburgh, United Kingdom; Clinica Universidad de Navarra, Spain

## Abstract

**Introduction:**

Combined inhaled long-acting beta-agonists and corticosteroids (LABA+ICS) are costly. They are recommended in severe or very severe chronic obstructive pulmonary disease (COPD). They should not be prescribed in mild or moderate disease. In COPD ICS are associated with side-effects including risk of pneumonia. We quantified appropriateness of prescribing and examined the risks and costs associated with overuse.

**Methods:**

Data were extracted from the electronic and paper records of 41 London general practices (population 310,775) including spirometry, medications and exacerbations. We classified severity, assessed appropriateness of prescribing using the Global Initiative for Chronic Obstructive Lung Disease (GOLD) guidelines for 2009, and performed a sensitivity analysis using the broader recommendations of the 2011 revision.

**Results:**

3537 patients had a diagnosis of COPD. Spirometry was recorded for 2458(69%). 709(29%) did not meet GOLD criteria. 1749(49%) with confirmed COPD were analysed: 8.6% under-treated, 38% over-treated. Over-prescription of ICS in GOLD stage I or II (n=403, 38%) and in GOLD III or IV without exacerbations (n=231, 33.6%) was common. An estimated 12 cases (95%CI 7-19) annually of serious pneumonia were likely among 897 inappropriately treated. 535 cases of overtreatment involved LABA+ICS with a mean per patient cost of £553.56/year (€650.03). Using the broader indications for ICS in the 2011 revised GOLD guideline 25% were still classified as over-treated. The estimated risk of 15 cases of pneumonia (95%CI 8-22) in 1074 patients currently receiving ICS would rise by 20% to 18 (95%CI 9.8-26.7) in 1305 patients prescribed ICS if all with GOLD grade 3 and 4 received LABA+ICS.

**Conclusion:**

Over-prescription of ICS in confirmed COPD was widespread with considerable potential for harm. In COPD where treatment is often escalated in the hope of easing the burden of disease clinicians should consider both the risks and benefits of treatment and the costs where the benefits are unproven.

## Introduction

Chronic Obstructive Pulmonary Disease (COPD) places a significant burden on health services. In the UK, as in many countries, most COPD management takes place in primary care[1-3]. However, the variable quality of spirometry in primary care and the appropriateness of COPD prescribing have been questioned[2,4,5]. Reflecting the hope that inhaled corticosteroids (ICS) may improve outcomes, particularly in severe disease[6], high rates of ICS prescribing for COPD have been reported in many countries, raising concerns about over-prescribing[1-3,7]. 

The effectiveness of ICS (normally prescribed in combination with long-acting beta-agonists (LABA)) in the prevention of exacerbations (in frequent exacerbators with more severe COPD) is established: their role in ameliorating COPD symptoms is unproven. Early hopes that they would improve quality of life (QoL) have not been confirmed[8]. Although statistically significant improvements in QoL scores have been shown in some large trials of combined ICS and LABA, none has shown an improvement reaching the minimal clinically important difference (MCID) of the instrument used[9-16]. In only three trials has the upper limit of the 95% confidence interval of the improvement in intervention group patients reached the MCID[11,14,15]. Disappointed in the lack of evidence [6,8] Sin and Man in 2010 made the questionable suggestion that “A more plausible (and simple) explanation (for the high worldwide sales of combined ICS/LABA) is that clinicians (and patients) use ICS-based therapy for COPD because they work”.[8] Discussion of the role of ICS in symptom management of COPD was notable by its absence in the recent Lancet review by Rabe and Wedzicha[17]. There is no evidence to support the use of ICS as mono-therapy or in combination with LAMA at any severity level of COPD.

Guidelines have adopted different positions in the face of equivocal evidence supporting ICS use for COPD symptoms. The UK National Institute for Health and Clinical Excellence (NICE) revised its COPD guideline in 2010 and included the advice that “in people with stable COPD and an FEV1 ≥ 50% predicted who remain breathless consider LABA and ICS in a combination inhaler”.[18] More cautiously the American College of Physicians’ clinical guideline on COPD states: “the evidence is insufficient to support a strong recommendation for the broad use of combination therapy, and clinicians will need to weigh the potential benefits and harms on a case by case basis.” In contrast, the Global Initiative for Chronic Obstructive Lung Disease (GOLD) guideline 2009 (current at the time of our data collection) recommended ICS to prevent exacerbations in patients with severe disease and a history of frequent exacerbations. The 2011 revised GOLD guideline now explicitly regards all patients with severe/very severe COPD as at risk of exacerbations and thus eligible for treatment with ICS/LABA regardless of their past exacerbation status[19]. Classification of severity was changed from four stages (I-IV) to four grades (1-4), using the same spirometry thresholds, but adding symptom-based estimates of severity. The guideline now recommends LABA+ICS for all patients in grades 3-4 on the basis that their risk of exacerbation is higher. It acknowledges the risk of pneumonia in COPD patients prescribed ICS but does not quantify this risk. Until the TORCH trial (2007) highlighted the risk of pneumonia, ICS were generally considered safe from serious side effects. Since then evidence of the risk of pneumonia and fractures in people with COPD on ICS has been growing[13,20-22]. Singh et al estimated the relative risk of severe pneumonia in COPD treated with combined LABA+ICS when compared to LABA alone to lie between 1.46 (CI 1.26-1.69) and 1.56 (CI 1.4-1.74). NICE estimated the annual number needed to harm (NNH) for severe pneumonia as a consequence of ICS use in COPD as 60 to 72 people[18,22]. The total cost of combined LABA+ICS in the UK is more than any other drug. In 2010, £497,665,559 (€584,469,239) was spent on combination inhalers in England, a 7% increase on the previous year (NHS Business Authority, Freedom of Information Request, 110407 Booth 515237, 7 April 2011). Much of this ICS/LABA prescribing is for COPD where the equivocal evidence, diverse interpretations, and changing recommendations in guidelines could lead to considerable confusion and inappropriate prescribing. This study aimed to examine at patient level, in a population of more than 300,000, the rate of prescribing of ICS for COPD, the adherence of that prescribing to international guidelines, and its impact on risk and cost in general practice. 

### Materials and Method

#### Ethics statement

Ethical approval was obtained from the South East Research Ethics Committee, REC reference 09/H1102/19. All patient data were anonymised prior to provision. No patient identifiable data were retained by the research team. 

We stratified 98 practices in the London boroughs of Lambeth and Southwark by list size and by socio-economic status using the 2007 Index of Multiple Deprivation (IMD) score derived from each practice’s postcode. IMD score is based on national census and local authority data, and reflects deprivation specific to a geographical area[23]. IMD scores in 2007 ranged nationally from 0 (least deprived) to 86 (most deprived). We invited 51 practices at random to participate. Practice characteristics were obtained from the NHS Information Centre Quality and Outcomes Framework database[24]. An administrator, employed by practices but trained by the research team, obtained data on all patients on the practice COPD registers using electronic and hand searches of records between December 2009 and November 2010. Data were provided in anonymised form. Participating practices received service support costs from The UK Primary Care Research Network. 

We collected demographic information (age, gender, ethnicity and postcode) and clinical data related to COPD diagnosis and management (including spirometry, inhaled and oral drug prescriptions in the last year, smoking history, occurrence of COPD exacerbations, hospital admissions, prescription of oral prednisolone for COPD in the last two years, and data on co-morbidities). We used patients’ postcodes to calculate patients’ IMD scores. 

#### Spirometry

Spirometry was accepted if FEV_1_ (forced expiratory volume in one second), FVC (forced vital capacity), height, date of birth and gender were available. These enabled GOLD severity grading. Biologically “implausible” values of FEV_1_ or FVC were excluded. Patients whose spirometry met criteria for a diagnosis of COPD (FEV_1_/FVC ratio <70%) were categorised into severity stages according to GOLD classification using FEV_1_ and history of exacerbations (Table 1). We accepted at least one exacerbation requiring oral steroids or hospital admission for COPD as evidence of risk of repeated exacerbations. We did this for three reasons: prescribing decisions might have taken into account similar events not explicitly noted in the medical record; LABA+ICS prescriptions may have been initiated in hospital clinics or during hospital admissions without recording the indication; and retrospective identification of the date of introduction of ICS and the frequency of exacerbation or admission in the preceeding three years was complex. This decision means we may have underestimated the over-prescribing of ICS. 

**Table 1:** Treatments considered acceptable at each GOLD stage (2009 update).

GOLD STAGE	FEV_1_ % predicted	No treatment*	SAMA/SABA**	LAMA/LABA	ICS***
I (mild)	≥80%	Yes	Yes	No	No
II (moderate)	50%≤FEV_1_<80%	Yes	Yes	Yes	No
III (severe)	30%≤FEV_1_<50%	Yes	Yes	Yes	No
IV (very severe)	<30%	Yes	Yes	Yes	No
III or IV with exacerbations	-	No	Yes	Yes	Yes

^*^ Without access to current symptom data (eg breathlessness) absence of treatment was considered appropriate in patients at all severity stages provided they had no exacerbations.

^**^ SAMA/SABA Short-acting muscarinic or beta_2_-agonist; LAMA/LABA Long-acting muscarinic or beta_2_-agonist; ICS Inhaled cortico-steroid

^***^ Treatment with LABA + ICS or ICS alone was acceptable for any patients with a diagnosis of asthma or history of asthma

Characteristics of patients with spirometry-confirmed COPD were compared using logistic regression to those whose COPD was not confirmed. Allowance for clustering within practices was made using the Huber-White robust variance estimator[25]. Quality of treatment was only analysed in patients with spirometry confirmed diagnosis of COPD. Data were analysed using STATA Version 11 (Statacorp, Texas). 

### Drug management and analysis

Inhaled drug treatment was assessed against GOLD (2009 update) according to the patient’s severity (Table 1)[26]. Patients were classified as under-treated, appropriately treated or over-treated for GOLD stage. In the absence of reliable symptom data, we accepted absence of treatment as appropriate in patients with no recorded exacerbations at any severity stage although we recognised that this may have represented under-treatment of symptoms. In line with asthma guidelines ICS were considered appropriate in patients with asthma, or a history of asthma, irrespective of their GOLD stage. Assessment of possible under-treatment with lower dose ICS (beclomethasone or budesonide in single drug inhalers) in severe or very severe COPD with exacerbations was complex due to the difficulty in counting total doses of these steroids prescribed over the study period. As less than 2% of these patients were on these lower dose steroid inhalers there may have been minor under-estimation of under-treatment in these patients. 

Predictors of over-treatment were sought using univariate and multiple logistic regression using the Huber-White robust variance estimator to account for clustering[25]. 

### Risk of pneumonia

Risk of pneumonia was assessed in all patients treated with ICS, and in those judged to be receiving ICS inappropriately. We used estimates of the relative risk of pneumonia associated with ICS treatment from the meta-analysis in the NICE 2010 guideline examining LABA+ICS versus LABA[18]. NICE estimated the relative risk (RR) for serious pneumonia in COPD as 1.46 (95%CI 1.26-1.69) for treatment with LABA+ICS compared to LABA[18]. A baseline event rate for serious pneumonia of 30 per 1000 person-years was used, as reported by Singh et al.[27] The annual number needed to harm (NNH) based on these figures was 72.4 (95% CI 48.3-128.2).

### Cost analysis

Costs of prescribed respiratory drugs in England in 2010 were provided by the NHS Business Services Authority in response to a Freedom of Information request (NHS Business Authority, Freedom of Information Request, 110407 Booth 515237, 7 April 2011). We obtained costs associated with prescription of all forms of LABA+ICS for England, London, and Lambeth and Southwark Primary Care Trusts (Primary Care Trusts are freestanding NHS bodies responsible for delivering health care and health improvements in their locality). We also obtained costs for the ten most expensive prescribed drugs by net ingredient cost for the same geographical areas with which we could compare the relative cost of respiratory drugs. Mean costs of inhaled respiratory drugs were estimated by applying the average monthly cost of the dose recommended in the British National Formulary (BNF) in a CFC-free single or combination preparation for each drug e.g. for ICS in a combination inhaler the mean cost of one month’s treatment with salmeterol and fluticasone (50mcg/500mcg twice daily via Evohaler® or Accuhaler®), and formoterol and budesonide (12mcg/400mcg twice daily via Turbohaler®) was taken[28]. Ranges for costs were calculated using the lowest and highest prices of the different available drugs.

### Sensitivity analysis

In 2011, 9 months after data collection was completed, the GOLD guideline was revised to incorporate broader indications for ICS prescribing. To evaluate the impact of the revised GOLD guideline on the assessment of rates of under-treatment and over-treatment, the predictors of overtreatment, and the risk of pneumonia, we carried out a sensitivity analysis replacing the 2009 GOLD guideline treatment definitions (Table 1) with the revised GOLD 2011 definitions (Supplementary information – Table S1)[19]. The revised analysis is presented in supporting information labeled Tables S1-S4 and Figure S1.

## Results

### Practice characteristics

**Figure 1:**
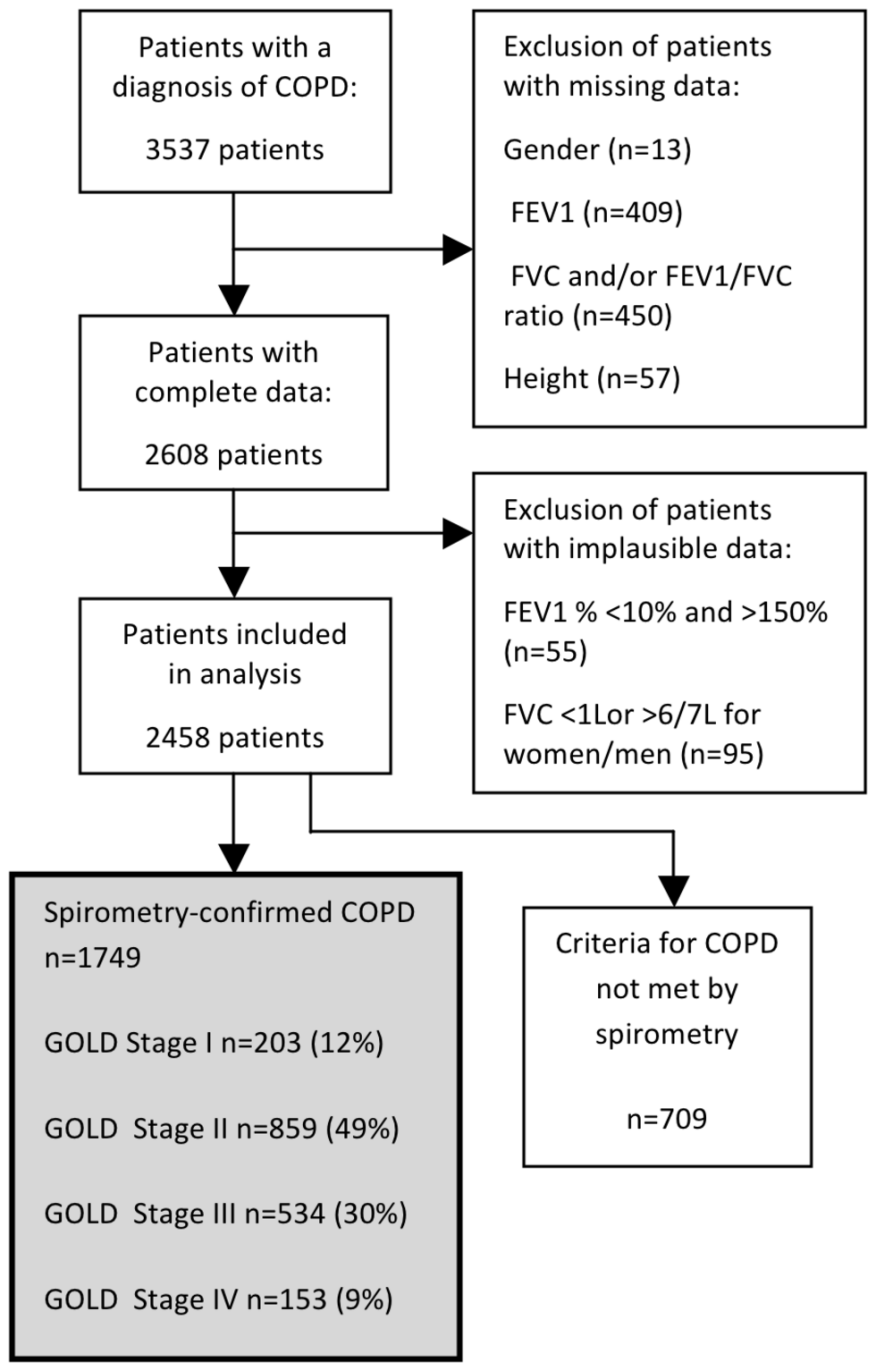
Identification of subjects and categorization by diagnosis and severity (GOLD stage) in 41 practices.

Data were obtained from 41 (80%) general practices serving a population of 310,775. There were no significant differences between the 41 participating and 10 non-participating practices in size, demographic and quality indicator characteristics (based on NHS Information Centre Quality and Outcomes Framework), and prevalence of COPD. 3537 (1.14%) patients with a diagnosis of COPD were identified. After removing records with incomplete or implausible spirometry, data from 2458 patients were used in the analysis of treatment appropriateness (Figure 1).

The characteristics of participating practices are shown in Table 2. 

**Table 2:** Practice characteristics.

	**Mean**	**SD**
Practice list size (n)	7998	4766
Index of Multiple Deprivation Score (IMD) 2007	33.1	9.9
COPD QOF* points awarded 2009/10 (% of available total)**	92.9%	15.6%
Overall QOF* points awarded 2009/10 (% of available total)***	92.0%	4.9%
Practice prevalence of COPD	1.03%	0.44%
Proportion of list >45 years (%)	28.6%	5.3%
Sex (% male)	50.9%	3.4%
Training practices† (%)	12 (31.58%)	-
Proportions of patients treated in line with GOLD‡ (%) (practice means and SD)	58.1%	19.4%
Proportions of patients under-treated according to GOLD‡ (%) (practice means and SD)	11.9%	8.9%
Proportions of patients over-treated according to GOLD‡ (%) (practice means and SD)	39.2%	19.0%

^*^ Quality and Outcomes Framework element of NHS GP contract

^**^ National average: 95.8%, SD 12.6%

^***^ National average: 93.7%, 6.4 SD %

^†^ Practice in which a GP trainer and the whole practice have been approved for the purposes of postgraduate training of general practitioners

^‡^ GOLD guideline 2009 update

### Patient characteristics

Patients with spirometry-confirmed COPD were compared to those whose COPD was not confirmed (Table 3). The average IMD score in our study (and in the local population) lay within the most deprived quintile of the country. Patients with spirometry-confirmed COPD were older, had lower FEV_1_ (where available), fewer co-morbidities, and were more likely to be ex- or current smokers. Ethnicity data were available for 54.8%. 80.3% were white and 8.5% were black compared to 62.7% white and 25.83% black in the local population[29,30].

**Table 3:** Patient characteristics and treatment.

	**All patients (n=3537)**	**Patients with COPD confirmed by spirometry (n=1749)**	**Characteristics of patients with COPD confirmed by spirometry (n=1749) compared to those whose spirometry did not meet criteria for COPD (FEV_1_/FVC≥0.7)(n=709)**	
			**OR**	**95% CI**
**Gender (Male)**	1807 (51.23%)	944 (53.97%)	1.14	0.86-1.52
**Age at data collection (years)**	69.8 (12.0)	68.3 (10.8)	1.02	1.01-1.03†
**Index of Multiple Deprivation (IMD**)*	35.5 (9.2)	35.9 (8.8)	0.1	0.96-1.03
**FEV1 (l)**	-	1.39 (0.58)	0.27	0.22-0.33†
**FEV1 (% predicted)**	-	56.9% (20.2%)	0.96	0.95-0.96†
**Number of co-morbidities**	1.4 (1.2)	1.32 (1.1)	0.86	0.81-0.92†
**Diagnosis of depression (%)**	709 (21.3%)	311 (19.0%)	0.76	0.62-0.93†
Never-smoker**	320 (9.0%)	113 (6.5%)	0.53	0.32-0.87†
**Ex-Smoker**	1473 (41.8%)	755 (43.2%)	1.23	1.01-1.5†
**Current smoker**	1160 (32.9%)	606 (34.7%)	1.10	0.92-1.33
**Oral steroids in the previous 2 years**	866 (24.6%)	455 (26.0%)	1.44	1.19-1.74†
**Hospital admission for COPD in the previous 2 years**	295 (8.47%)	153 (8.8%)	1.48	1.11-1.99†
**Exacerbation in the previous 2 years**	456 (12.9%)	235 (13.4%)	1.44	1.14-1.82†
**Long-term oxygen therapy**	131 (3.7%)	60 (3.4%)	1.54	0.88-2.68
**Treatment with SABA**	2551 (72.3%)	1338 (76.5%)	1.56	1.29-1.89†
**Treatment with SAMA**	518 (14.7%)	290 (16.6%)	1.52	1.10-2.09†
**Treatment with LABA**	623 (17.7%)	351 (20.1%)	1.21	0.92-1.58
**Treatment with LAMA**	1359 (38.5%)	791 (45.2%)	2.07	1.63-2.64†
**Treatment with ICS**	1200 (34.0%)	628 (35.9%)	0.99	0.70-1.4
**Treatment with LABA + ICS combined**	1333 (37.8%)	705 (40.3%)	1.18	0.93-1.49
**Treatment with ICS (combination or single)**	2076 (58.9%)	1074 (61.41%)	1.12	0.89-1.41

SABA=short-acting beta_2_-agonist; SAMA = short-acting muscarinic antagonist; LABA = long-acting beta_2_-agonist; LAMA = long-acting muscarinic antagonist; ICS = inhaled corticosteroid

* Mean national IMD for 2007 was 21.7 (range 0.4-85.5); mean IMD for Lambeth and Southwark was 34.2 (range 7.1-58.9)

** Smoking status data were missing in 17% of patients

† Indicates a significant difference

### Diagnosis and disease severity

709 (28.8%) patients with complete spirometry records did not meet the COPD diagnostic criterion of an FEV_1_/FVC ratio <70%. GOLD stages of those with spirometry are shown in Figure 1. 

### Exacerbations

26% of patients with spirometry-confirmed COPD had at least one course of high-dose oral steroids (≥20mg prednisolone daily) in the previous two years, and 8.8% had been admitted to hospital with an exacerbation. Patients with confirmed COPD were more likely to have had oral steroids, had an exacerbation, been admitted to hospital with COPD, and have received treatment with SABA, SAMA or LAMA than patients without spirometric confirmation. 

### Drug treatment

**Figure 2:**
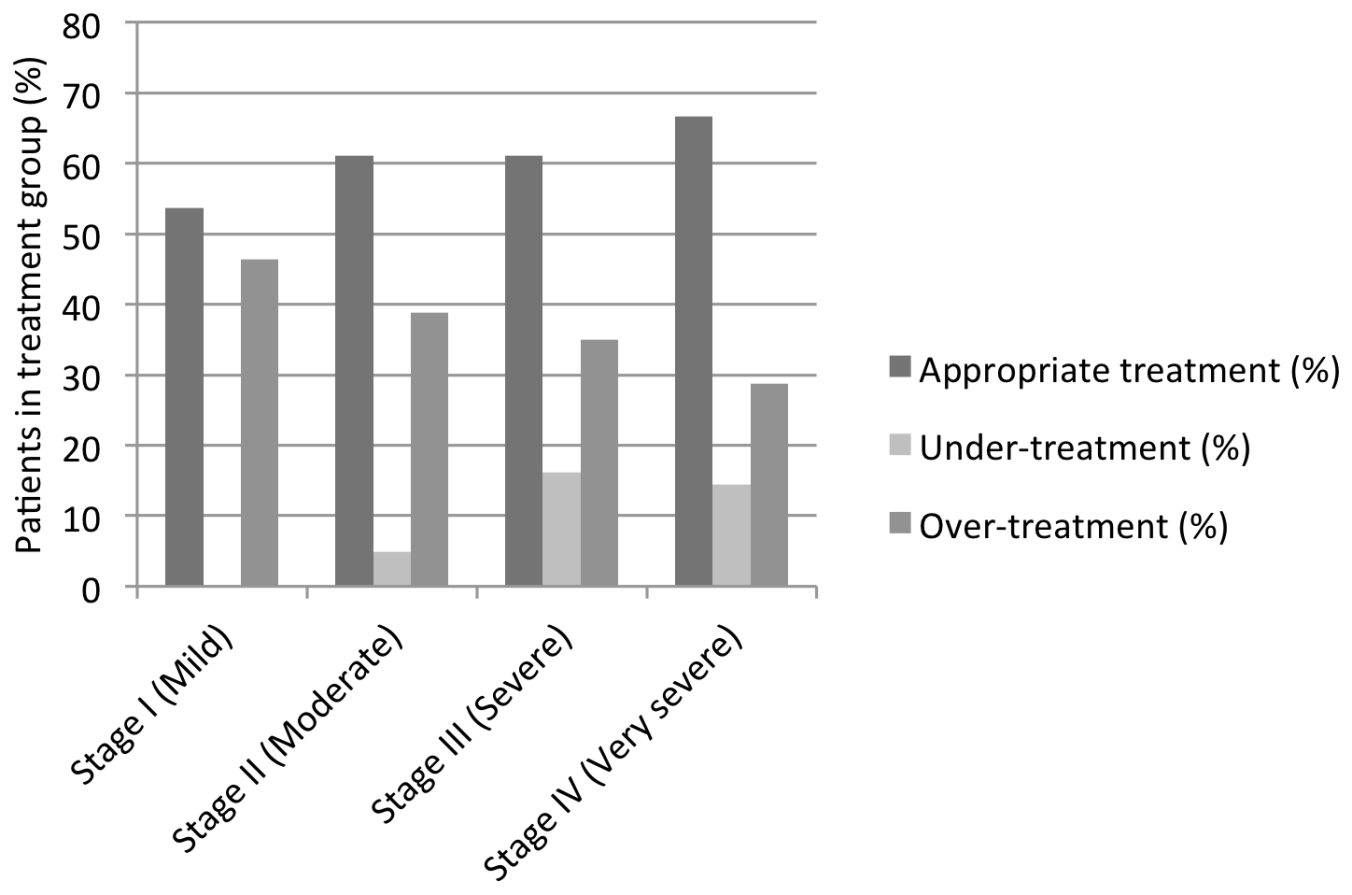
Proportion of patients with spirometric confirmation of diagnosis of COPD in each treatment classification by GOLD stage.

Inhaled medications were prescribed in line with 2009 GOLD guidelines for 59.8% patients with confirmed COPD. 8.6% were under-treated and 37.7% appeared to be over-treated for their GOLD stage (table 4). 106 patients were classed as both under- and over-treated as they were over-treated with ICS but under-treated without SABA and/or LABA. Over-treatment was more likely in mild patients and under-treatment increased with severity (Figure 2). Of the 659 patients classed as over-treated 634 (96.2%) were over-treated with ICS. Among the 469 patients without spirometry-confirmed COPD or a diagnosis of asthma, 238 (50.7%) were receiving ICS.

**Table 4:** Patients with spirometry-confirmed COPD: Treatment by GOLD stage (2009 update).

**GOLD** † **stage**	Treated in line with GOLD† recommendations (%)	Under-treated according to GOLD† recommendations (%)	Over-treated according to GOLD† recommendations (%)
**I - mild (n=203)**	109 (53.7%)	0 (0%)	94 (46.3%)
**II - moderate (n=859)**	509 (61.1%)	42 (4.89%)	334 (38.9%)
**III - severe** (**n=534**)*	326 (61.1%)	86 (16.1%)	187 (35.0%)
**IV - very severe (n=153)**	102 (66.7%)	22 (14.38%)	44 (28.8%)
**Total (n=1749)**	**1046 (59.8%)**	**150 (8.6%)**	**659 (37.68%)**

* Some patients were classed as both under and over-treated

† GOLD 2009 update

### Predictors of overtreatment

Predictors of over-treatment according to GOLD stage were sought using univariate and multivariate analysis (Table 5). Variables with the strongest univariate associations with over-treatment (Table 5) were MRC Dyspnoea Score (over-treatment less likely with more severe MRC score), GOLD stage (over-treatment less likely in GOLD stage III or IV), and exacerbations or hospital admissions for COPD in the previous two years (over-treatment less likely in patients who reported an exacerbation requiring oral steroids or a hospital admission for COPD). Predictors of over-treatment in the multivariate analysis were an exacerbation of COPD in the last two years, IMD score and MRC score. 

**Table 5:** Relationship between overtreatment and patient characteristics in patients with a spirometry confirmed diagnosis of COPD: univariate and multiple logistic regression (using Huber-White robust variance estimator to allow for clustering). (GOLD 2009 update).

**Variable**	**Number overtreated (%)**	**OR (95% CI)**
*Age(years)*	659 (38%)	1.01 (1.00-1.01)
*Gender*		
Female	299 (37%)	1.0
Male	360 (38%)	1.04 (0.86-1.27)
*Index of Multiple Deprivation Score*	659 (38%)	1.02 (1.01-1.03)
*Smoking status*		
Never smoker	40 (35%)	1.0
Ex-smoker	307 (41%)	1.25 (0.81-1.93)
Smoker	240 (40%)	1.2 (0.83-1.72)
*GOLD Stage*		
Stage I or II (mild to moderate)	428 (40%)	1.0
Stage III or IV (severe or very severe)	231 (34%)	0.75 (0.59-0.95)
*MRC Dyspnoea Score*		
1-2	201 (37%)	1.0
3	184 (47%)	1.53 (1.17-2.00)
4	116 (42%)	1.22 (0.90-1.64)
5	28 (42%)	1.25 (0.70-2.35)
*Depression*		
No	529 (40%)	1.0
Yes	120 (39%)	0.94 (0.74-1.20)
*Exacerbation for COPD in <2 years*		
No	604 (40%)	1.0
Yes	55 (23%)	0.46 (0.3-0.71)
*Hospital Admission for COPD in <2 years*		
No	625 (39%)	1.0
Yes	34 (22%)	0.44 (0.3-0.66)
*Number of co-morbidities*		
0	167 (38%)	1.0
1-2	378 (37%)	0.95 (0.71-1.28)
≥3	114 (42%)	1.19 (0.81-1.76)
Multiple Logistic Regression*		
*MRC Dyspnoea Score*		
1-2	1.0	
3	1.66 (1.23-2.22)	
4	1.44 (1.04-2.00)	
5	2.08 (1.02-4.23)	
*COPD exacerbation in the last 2 years*	0.35 (0.18-0.68)	
*Index of Multiple Deprivation score*	1.02 (1.01-1.03)	

* Controlling for age, gender and GOLD stage. Pseudo R^2^ 0.0294. Describes how well (2.9%) the model performs when compared to a perfect prediction model

### Risk of pneumonia

Using the estimated NNH of 72.4, 29 cases (95%CI 16-43) of serious pneumonia were likely to have been observed among the 2076 taking ICS. Twelve cases (95% CI 7-19) were likely to have occurred in the 897 patients with or without confirmed COPD whom we assessed as being inappropriately treated with ICS.

### Cost of overtreatment

The mean monthly price for combined LABA+ICS at the time of the study was £46.13(€54.16) and for single preparation ICS inhalers was £12.76(€14.98). Of the 659 (37.68%) with confirmed COPD over-treated in this study 397 were prescribed combination inhalers and the remainder single preparation ICS. Based on the mean drug cost, the estimated cost of over-prescribing ICS in these patients was £21,657(€25,425) per month, or £259,881(€305,102) per year (range per year: £195,023-£357,530)(€ 228,933-€ 419,696). In the 709 patients in whom spirometry criteria for COPD were not met and who did not have a diagnosis of asthma 238 (33.6%) were receiving ICS (138 on combination inhaler and 100 on single preparation). The estimated cost of prescribing ICS in these patients each year was £91,244(€107,119)(range per year: £68,108(€79,968)-£125,244(€147,078)).

If we assume that the severity and prescribing patterns of the patients whose treatment appropriateness we were unable to analyse due to incomplete spirometry data were the same as those included in the study, the overall cost of overprescribing would have been £505,260(€593,275) per year in the 41 practices (£12,323(€14,470) per practice).

### Sensitivity analysis using the 2011 changes in the GOLD guideline

The results of the sensitivity analysis which repeated all the analyses are given in the attached supporting information, Tables S1-S4. As a result of the application of the 2011 GOLD Guidelines 231 severe/very severe COPD patients (GOLD Stages III and IV) originally classified as over-treated (they were taking inhaled corticosteroids and had not had an exacerbation in the previous year) were reclassified as appropriately treated (Supporting information - Table S1). This would reduce the overall proportion of over-treated patients among those with spirometry confirmed COPD from 38% to 24% (Supporting information – Table S3), and reduce the practice mean proportion of patients over-treated to 17.1% (Supporting information –Table S2). 203 severe/very severe COPD patients (GOLD Stages III and IV) originally classified as appropriately or under-treated because they had not had inhaled corticosteroids were reclassified as under-treated. Overtreatment of COPD was more likely in patients in GOLD grade 1 compared to GOLD grade 2 (in which overtreatment with SABA, LAMA, and LABA was no longer possible according to GOLD 2011 revision) when controlling for exacerbations, hospitalisation, MRC score, IMD score, smoking history, and other co-morbidities, (multiple logistic regression - OR 1.5, 95% CI 1.06-2.2; n=794) (Supplementary information - Table S4). No other variables were predictive of overtreatment in multivariate analysis. If all patients with GOLD grade 3 and 4 received LABA+ICS the estimated cases of pneumonia would rise by 20% from 15 (95% CI 8-22) cases in the 1074 patients with confirmed COPD currently receiving ICS to 18 (95% CI 9.8-26.7) cases each year in the 1305 patients who would qualify for ICS according to the updated guidelines 

## Discussion

Over-treatment of COPD with inhaled corticosteroids was widespread in this large study of COPD management. Of patients who had a diagnosis of COPD confirmed by spirometry, 38% were over-treated according to the GOLD guidelines current at the time of prescribing. Excluding people with asthma, in 96% of these cases over-treatment was with ICS, resulting in an estimated 12 additional cases of severe pneumonia every year[18]. Under-treatment was seen in approximately 9% of patients, though this may be an underestimate due to the absence of information on current symptoms such as breathlessness, the only treatment indication in mild and moderate COPD.

We estimated the annual cost of over-prescribing in this study population to be £505,260 (€593,373) based on mean prices for ICS[28]. If similar levels of over-prescribing were seen throughout England the unnecessary cost would have been about £102million (€119.8million) per year; this equates to more than a fifth of actual spending on combination inhalers in the year of the study. 

The 2011 revision of the GOLD guidelines changed retrospectively how these data might be interpreted now. Estimates of overtreatment would fall from 38% to 24% and the annual cost in England of this over-treatment would be £67 million (€79 million). Appropriate treatment would rise from 60% to 68% a smaller difference because some of those patients who were over-treated with ICS were also under-treated by not having had long-acting bronchodilators prescribed. If the revised guideline prescribing indications were adhered to consistently, the estimate of the risk of pneumonia in COPD patients as a result of ICS prescribing would rise by 20%, raising important issues about the balance of risks and benefits especially in patients with severe and very severe COPD.

### Limitations and strengths

A key issue in this study is the accuracy, completeness, and reliability of routinely collected data extracted from primary care records. All participating practices used computerised prescribing so the prescription recorded was almost certainly the prescription issued, although patients may not have presented every prescription at a chemist, nor used the drug that had been dispensed. 

Spirometry results are essential to clinical treatment decisions in COPD and should be recorded in the patient’s record. The 2009 GOLD indications for prescribing ICS, which were based on GOLD stage (% predicted FEV1) together with history of exacerbations, have been revised and are now based mainly on spirometry results in the 2011 GOLD update. Bronchodilator prescribing is more difficult to judge because objective recording of symptom status, the main indication, is inconsistent[31]. We erred on the side of the prescriber assuming absence of symptom recording meant absence of symptoms and that bronchodilator treatment was not indicated. This may have resulted in an underestimate of under-prescribing. We may also have under-estimated over-prescribing according to 2009 GOLD by defining the indication for ICS in severe and very severe COPD as one exacerbation requiring oral steroids or admission rather than three exacerbations in the three years. This indication was removed in the 2011 GOLD guidelines. 

Participating practices were recruited from two adjacent localities. However, participating practices were stratified by size and socio-economic deprivation and randomly invited. They served 62% of the population in the two study boroughs. We found no differences between participating and non-participating practices. The prescribing rates for combination inhalers in these two boroughs were similar to national rates, so it is unlikely that our findings were significantly different to overall prescribing patterns in England.

We based our assessment of ICS appropriateness on spirometry in the previous two years. This reflects the evidence base as all drug trials in COPD classify participants by spirometry. Until trials of ICS in COPD are conducted using broader assessment criteria, spirometry without exacerbation history will remain the main arbiter of treatment suitability. We did not collect data on pneumonia rates in COPD patients because, despite the inherent inaccuracy of diagnosing pneumonia without a chest x-ray which is rarely performed in primary care, it was beyond the means of the study to estimate the rate of occurrence of pneumonia in relation to duration of exposure to combination inhalers. 

### Interpretation in relation to existing literature

A significant proportion of patients with a diagnosis of COPD had not had their spirometric status confirmed and recorded. In nearly a third that had had spirometry the record did not support a diagnosis of COPD, comparable to findings in other studies[1-3]. These diagnoses may have been made on the basis of symptoms for which spirometry was not yet obtained, or may have been the result of coding based on an incorrect hospital discharge summary or radiology report. A number of patients without spirometry confirmed COPD had a history of hospital admissions or oral steroid prescription for COPD. Some of this group may have had COPD but did not have diagnostic confirmation due to poorly conducted spirometry, or due to failure to attend for spirometry once the exacerbation had resolved. Whilst the causes of this poor coding may be multifactorial, there is on-going concern that a significant proportion of patients are being prescribed high dose treatments with potential side effects with no confirmed diagnosis.

Over-prescribing of ICS in COPD has been reported in a number of countries[1,2,32,33]. Factors underlying this may be the perceived similarity of COPD and asthma, the common occurrence of the two diseases together, and a hope that steroids could reduce the impact of symptoms in COPD. ICS in the low or moderate doses recommended in asthma, cause few, mainly topical, side effects, so the potential benefits may outweigh any disadvantages[34]. In COPD ICS are used in higher doses with a corresponding increase in side effects. Critical assessment of the risk/benefit ratio for an individual patient would suggest targeting treatment at those with severe disease and at greatest risk of frequent exacerbations. 

We used the 2009 international GOLD guidelines to assess treatment[26], which were consistent with the 2004 NICE guidance current at the time of data collection. Since then guidelines have diverged in their interpretation of the equivocal data on benefits of ICS in reducing symptoms. GOLD still defines the only indication for ICS use in COPD as the prevention of exacerbations, though it now defines all patients with severe/very severe COPD as being at risk. In contrast, the updated UK NICE guideline in 2010 broadened the indication for ICS in COPD to persistent breathlessness in mild and moderate disease [18], a change likely to encourage yet more prescribing of ICS. Unsurprisingly, it appears from this study that primary care clinicians, faced with patients with on-going symptoms will often err on the side of prescribing with the attendant increased risks and escalating costs to health services. 

### Conclusion

The majority of COPD patients in our study population were treated appropriately according to international guidelines. A significant proportion was over-treated with high-dose inhaled corticosteroids – costly drugs that pose significant risks. The situation is not helped when guidelines provide conflicting and changing interpretations of equivocal data. A balanced assessment (ideally from a source unaffected by conflicts of interest) would help clinicians to weigh up the risks and benefits of treatment for a progressive condition such as COPD where treatment is often escalated in the hope of easing the increasing burden of disease. 

## Supporting Information

Table S1 **Treatments considered acceptable at each GOLD stage – based on GOLD revised 2011.**
(DOCX)

Table S2 **Practice characteristics based on Gold Revised 2011.**
(DOCX)

Table S3 **Patients with spirometry-confirmed COPD: Treatment by GOLD Revised 2011.**
(DOCX)

Table S4 **Relationship between overtreatment and patient characteristics in patients with spirometry confirmed Gold Grade 1 or Grade 2 COPD: univariate and multiple logistic regression (using Huber-White robust variance estimator to allow for clustering). (based on Gold Revised 2011).**
(DOCX)

Figure S1 **Proportion of patients with spirometry confirmed COPD in each treatment classification by GOLD stage.** (TIF)
