# Supplementary material for: Overtreatment of COPD with Inhaled Corticosteroids - Implications for Safety and Costs: Cross-Sectional Observational Study
Source: PLoS One. 2013 Oct 23;8(10):e75221. doi: 10.1371/journal.pone.0075221 (PMC3806778; doi:10.1371/journal.pone.0075221)
Supplement: Table S1 — Treatments considered acceptable at each GOLD stage – based on GOLD revised 2011. (DOCX) [file pone.0075221.s001.docx]

**Table S1: Treatments considered acceptable at each GOLD stage – based on GOLD revised 2011**

| GOLD STAGE | FEV_1_ % predicted | No treatment* | SAMA/SABA** | LAMA/LABA | ICS*** |
| --- | --- | --- | --- | --- | --- |
| I (mild) | ≥80% | Yes | Yes | No | No |
| II (moderate) | 50%≤FEV_1_<80% | Yes | Yes | Yes | No |
| III (severe) | 30%≤FEV_1_<50% | Yes | Yes | Yes | Yes |
| IV (very severe) | <30% | Yes | Yes | Yes | Yes |

*Without access to current symptom data (eg breathlessness) absence of treatment was considered appropriate in patients at all severity stages provided they had no exacerbations.

**SAMA/SABA Short-acting muscarinic or beta_2_-agonist; LAMA/LABALong-acting muscarinic or beta_2_-agonist; ICS Inhaled cortico-steroid

*** Treatment with LABA + ICS or ICS alone was acceptable for any patients with a diagnosis of asthma or history of asthma
