# Supplementary material for: Overtreatment of COPD with Inhaled Corticosteroids - Implications for Safety and Costs: Cross-Sectional Observational Study
Source: PLoS One. 2013 Oct 23;8(10):e75221. doi: 10.1371/journal.pone.0075221 (PMC3806778; doi:10.1371/journal.pone.0075221)
Supplement: Table S2 — Practice characteristics based on Gold Revised 2011. (DOCX) [file pone.0075221.s002.docx]

**Table S2: Practice characteristics based on Gold Revised 2011**

|  | **Mean** | **SD** |
| --- | --- | --- |
| Practice list size (n) | 7998 | 4766 |
| Index of Multiple Deprivation Score (IMD) 2007 | 33.1 | 9.9 |
| COPD QOF* points awarded 2009/10 (% of available total)** | 92.9% | 15.6% |
| Overall QOF* points awarded 2009/10 (% of available total)*** | 92.0% | 4.9% |
| Practice prevalence of COPD | 1.03% | 0.44% |
| Proportion of list >45 years (%) | 28.6% | 5.3% |
| Sex (% male) | 50.9% | 3.4% |
| Training practices† (%) | 12 (31.58%) |  |
| Proportions of patients treated in line with GOLD (%) (practice means and SD) | 82.1% | 10.6% |
| Proportions of patients under-treated according to GOLD (%) (practice means and SD) | 11.9% | 8.9% |
| Proportions of patients over-treated according to GOLD (%) (practice means and SD) | 17.1% | 10.7% |

* Quality and Outcomes Framework element of NHS GP contract(24)

**National average: 95.8%, SD 12.6%

***National average: 93.7%, 6.4 SD %

†Practice in which a GP trainer and the whole practice have been approved for the purposes of postgraduate training of general practitioners
