# Supplementary material for: Overtreatment of COPD with Inhaled Corticosteroids - Implications for Safety and Costs: Cross-Sectional Observational Study
Source: PLoS One. 2013 Oct 23;8(10):e75221. doi: 10.1371/journal.pone.0075221 (PMC3806778; doi:10.1371/journal.pone.0075221)
Supplement: Table S3 — Patients with spirometry-confirmed COPD: Treatment by GOLD Revised 2011. (DOCX) [file pone.0075221.s003.docx]

**Table S3: Patients with spirometry-confirmed COPD: Treatment by GOLD Revised 2011**

| **GOLD grade** | Treated in line with GOLD recommendations (%) | Under-treated according to GOLD recommendations (%) | Over-treated according to GOLD recommendations (%) |
| --- | --- | --- | --- |
| **1- mild (n=203)** | 109 (53.7%) | 0 (0%) | 94 (46.3%) |
| **2 - moderate (n=859)** | 509 (59.3%) | 42 (4.89%) | 334 (38.9%) |
| **3 - severe (n=534)*** | 448 (83.9%) | 86 (16.1%) | 0 (00%) |
| **4 - very severe (n=153)** | 131 (85.6%) | 22 (14.38%) | 0 (0%) |
| **Total (n=1749)** | **1,197 (68.44%)** | **150 (8.6%)** | **428 (24.5%)** |

*Some patients were classed as both under and over-treated
