# Supplementary material for: Overtreatment of COPD with Inhaled Corticosteroids - Implications for Safety and Costs: Cross-Sectional Observational Study
Source: PLoS One. 2013 Oct 23;8(10):e75221. doi: 10.1371/journal.pone.0075221 (PMC3806778; doi:10.1371/journal.pone.0075221)
Supplement: Table S4 — Relationship between overtreatment and patient characteristics in patients with spirometry confirmed Gold Grade 1 or Grade 2 COPD: univariate and multiple logistic regression (using Huber-White robust variance estimator to allow for clustering). (based on Gold Revised 2011). (DOCX) [file pone.0075221.s004.docx]

**Table S4. Relationship between overtreatment and patient characteristics in patients with spirometry confirmed Gold Grade 1 or Grade 2 COPD: univariate and multiple logistic regression (using Huber-White robust variance estimator to allow for clustering). (based on Gold Revised 2011)**

| **Variable** | **Number overtreated (%)** | **OR (95% CI)** |
| --- | --- | --- |
| *Age(years)* | 428 (40%) | 1.01 (0.99-1.02) |
| *Gender* |  |  |
| Female | 217 (51%) | 1.0 |
| Male | 211 (55%) | 0.99 (0.78-1.27) |
| *Index of Multiple Deprivation Score* | 428 (40%) | 0.99 (0.98-1.01) |
| *Smoking status* |  |  |
| Never smoker | 28 (37%) | 1.0 |
| Ex-smoker | 206 (45%) | 1.38 (0.83-2.28) |
| Smoker | 150 (40%) | 1.1 (0.66-1.84) |
| *GOLD Grade* |  |  |
| Grade 1 (mild) | 428 (65.0%) | 1.0 |
| Grade 2 (moderate) | 231 (35.1%) | 0.74 (0.54-1.004) |
| *MRC Dyspnoea Score* |  |  |
| 1-2 | 152 (37%) | 1.0 |
| 3 | 122 (52%) | 1.84 (1.33-2.6) |
| 4 | 63 (47%) | 1.49 (1.004-2.21) |
| 5 | 11 (55%) | 2.05 (0.83-5.06) |
| *Depression* |  |  |
| No | 335 (42%) | 1.0 |
| Yes | 86 (44%) | 1.08 (0.79-1.48) |
| *Exacerbation for COPD in <2 years* |  |  |
| No | 373 (39%) | 1.0 |
| Yes | *55* (59%) | 2.25 (1.46-3.5) |
| *Hospital Admission for COPD in <2 years* |  |  |
| No | 394 (39%) | 1.0 |
| Yes | 34 (64%) | 2.8 (1.57-4.97) |
| *Number of co-morbidities* |  |  |
| 0 | 98 (40%) | 1.0 |
| 1-2 | 254 (40%) | 1.02 (0.76-1.38) |
| ≥3 | 76 (41%) | 1.03 (0.7-1.52) |
| **Multiple Logistic Regression*** |  |  |
| *MRC Dyspnoea Score* |  | |
| 1-2 | 1.0 | |
| 3 | 1.88 (1.35-2.61) | |
| 4 | 1.5 (1.01-2.23) | |
| 5 | 2.14 (0.86-5.32) | |
| *Gold stage 1* |  | |
| 1 | 1 | |
| 2 | 1.52 (1.06-2.18) | |

*Controlling for age and gender. Pseudo R^2^ 0.026. Describes how well (2.6%) the model performs when compared to a perfect prediction model
