# Supplementary figures and images for: Overtreatment of COPD with Inhaled Corticosteroids - Implications for Safety and Costs: Cross-Sectional Observational Study
Source: PLoS One. 2013 Oct 23;8(10):e75221. doi: 10.1371/journal.pone.0075221 (PMC3806778; doi:10.1371/journal.pone.0075221)

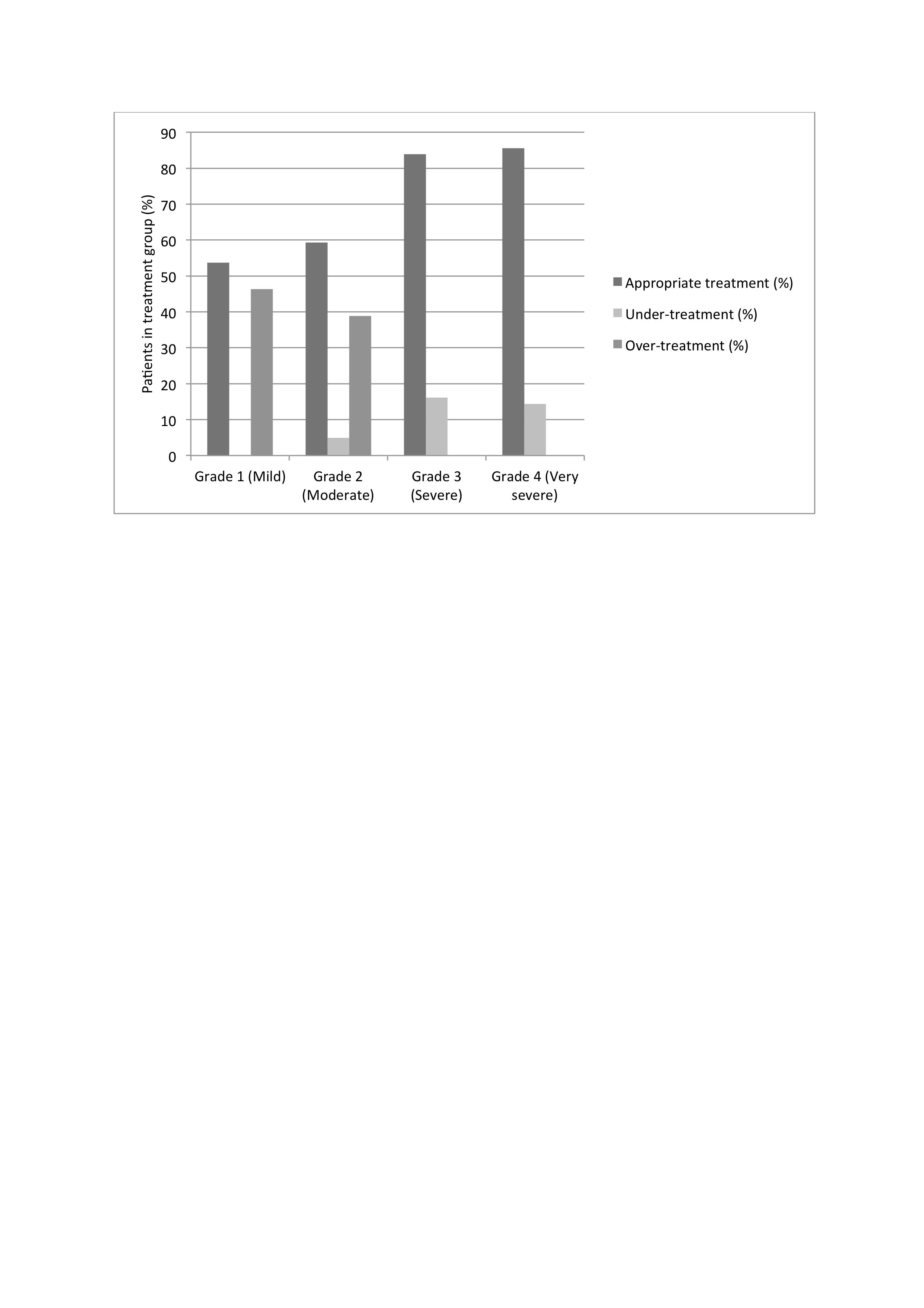

Supplement: Figure S1 — Proportion of patients with spirometry confirmed COPD in each treatment classification by GOLD stage. (TIF) [file pone.0075221.s005.tif]
